# Mid-term outcome of catheter ablation of idiopathic non-outflow tract ventricular arrhythmias

**DOI:** 10.1186/s12872-023-03702-0

**Published:** 2024-01-08

**Authors:** Dian Cheng, Jinbo Yu, Kanghui Chen, Xiaorong Li, Fengxiang Zhang, Weizhu Ju, Hongwu Chen, Gang Yang, Mingfang Li, Kai Gu, Xuecheng Wang, Xin Xie, Yizhang Wu, Jian Zhou, Xiaoqian Zhou, Pipin Kojodjojo, Bing Yang, Minglong Chen

**Affiliations:** 1 Department of Cardiology, Shanghai East Hospital, Tongji University School of Medicine, 150 Jimo Road, Shanghai, 200120 People’s Republic of China; 2 Department of Cardiology, The First Affiliated Hospital of Nanjing Medical University, Nanjing, People’s Republic of China; 3 Department of Cardiology, Jiangsu Province Hospital of Chinese Medicine, Nanjing, People’s Republic of China; 4 Department of Cardiology, Ng Teng Fong General Hospital, Singapore, Singapore

**Keywords:** Catheter ablation, Non-outflow tract, Ventricular arrhythmias

## Abstract

**Background:**

Catheter ablation is recommended in patients with frequent and symptomatic ventricular arrhythmias (VAs) in an otherwise normal heart. Right or left outflow tract (OT) are the most common origins, and catheter ablation is highly effective with low complication rates. However, outcome of catheter ablation of VAs other than the OT (non-OTVAs) is limited. The aim of this single-center study was to assess the safety and mid-term outcome of catheter ablation for non-OTVAs.

**Method and Results:**

From 2013 to 2018, 251 patients who underwent catheter ablation for idiopathic non-OTVAs were enrolled and grouped according to the origins including His-Purkinje system (HPS, n = 108), papillary muscle / moderator band (PM/MB, n = 47), tricuspid annulus (TA, n = 70), and mitral annulus (MA, n = 26), 244 (97.2%) had acute elimination of VAs. The time of VAs recurrence of the single procedure was 1.69 (0.12,9.72) months, with 66% occurring within the first 3 months. The recurrence rate was significantly higher in the PM/MB group than in the TA (*p* = 0.025) and MA groups (*p* = 0.023). The single procedure success rate in all patients was 70.1%, in which 66.7%, 59.6%, 80%, and 76.9% were achieved in the HPS, PM/MB, TA, and MA groups, respectively (*p* = 0.284). After multiple procedures, the total success rate was 76.5% at the follow-up of 4.38 ± 2.42 years. The rate was significantly lower in the PM/MB group than in the TA group (*p* = 0.035). In subgroup analysis, no significant difference was observed in the recurrence rate of single procedure in patients with different VA origins within the PM/MB (log-rank test, *p* = 0.546).

**Conclusion:**

Despite a certain percentage of recurrences observed in the mid-term follow-up, catheter ablation remained feasible and effective for idiopathic non-OTVAs.

**Supplementary Information:**

The online version contains supplementary material available at 10.1186/s12872-023-03702-0.

## Introduction

Idiopathic premature ventricular contraction (PVC) and ventricular tachycardia (VT) mostly originate from the right or left ventricular outflow tract (OT), and radiofrequency catheter ablation (RFCA) has been demonstrated effective in the treatment of idiopathic OT-VAs [1, 2]. Meanwhile non-OT ventricular arrhythmias (VAs) arising from His-Purkinje system (HPS), papillary muscle (PM) / moderator band (MB), tricuspid annulus (TA), and mitral annulus (MA) are not rare [3]. Due to the complex anatomy and electrophysiological characteristics, catheter ablation for non-OTVAs can be challenging and is associated with a lower success rate when compared to OT-VAs [4, 5]. Most previous studies reported the electrocardiogram findings, electrophysiological characteristics and ablation strategies of VAs originating from the HPS, PM, TA, and MA [5–9]. With advances in catheter ablation technology, researchers have increasingly focused on the safety and efficacy of catheter ablation of non-OTVAs. However, the mid-term outcome of the catheter ablation of idiopathic non-OTVAs is lacking.

This study aimed to evaluate the mid-term follow-up of catheter ablation for idiopathic non-OTVAs originating in the HPS, PM/MB, TA, and MA. We also performed subgroup analyses of VAs originating from specific sites within the HPS and PM/MB to determine whether success rates vary according to the site of origin.

## Methods

### Patient selection

**Fig. 1:**
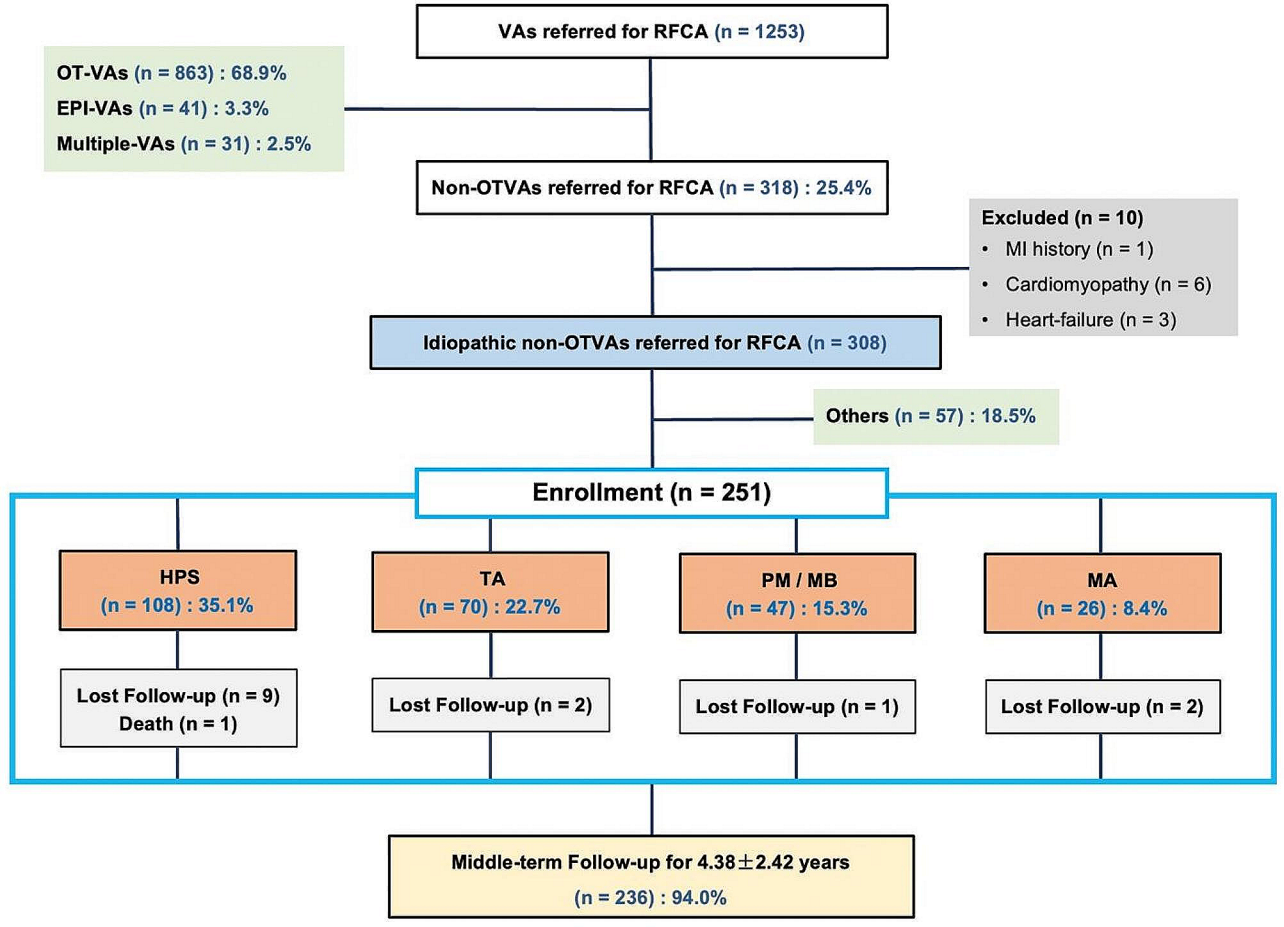
Flowchart of enrollment. VAs, ventricular arrhythmias; RFCA, radio frequency catheter ablation; OT, outflow tract; EPI, epicardial; MI, myocardial infarction; HPS, His-purkinje system; TA, tricuspid annulus; PM, papillary muscles; MB, moderator band; MA, mitral annulus

Among 1253 consecutive patients who underwent RFCA for VAs at the First Affiliated Hospital of Nanjing Medical University between October 2013 and August 2018, 251 (20%) patients with idiopathic VAs arising from the main sites of non-OT (including HPS, PM/MB, TA, and MA) were retrospectively enrolled. Echocardiography and exercise stress testing or coronary angiography demonstrated no evidence of structural heart disease in any patient. The exclusion criteria were as follows: (1) patients with structural heart disease; (2) patients with New York Heart Association (NYHA) class III/IV; (3) patients with a history of myocardial infarction and/or percutaneous coronary intervention within the last 6 months; (4) patients planned for heart transplantation. 57 patients originated from other rare sites of non-OT (including left or right ventricular free wall, apex and bottom, etc.) were not included (Fig. 1). Cases with origins of left para-hisian (n = 13) and right para-hisian (n = 11) areas were included in the MA group and TA group, respectively. The preoperative standard 12-lead ECG of patients showed monomorphic PVC or VT, and the origin of VAs was identified according to the successful ablation site. All eligible patients were provided written informed consent before the procedure, the study was undertaken after approval of the protocol by the Institutional Review Board.

### Electrophysiology study and catheter ablation

Antiarrhythmic drugs were discontinued for at least 5 half-lives. The procedures were performed under local anesthesia with guidance from the Carto (Biosense Webster, Diamond Bar, CA, USA) or Ensite mapping system (Abbott, St Paul, MN, USA). A 6-French (6 F) quadripolar and 6 F decapolar catheter (Abbott, St Paul, MN, USA or Biosense-Webster Inc., CA, USA) were advanced into the right ventricle apex and coronary sinus, respectively, via femoral veins. If clinical VAs failed to occur spontaneously, intravenous isoproterenol (2–5 µg/min) was infused, or a standard programmed ventricular stimulation protocol was performed. The origin was initially predicted based on the 12-lead ECG characteristics. A 4-mm- or 3.5-mm-tip irrigated catheter (ThermoCool SmartTouch or Navistar Thermocool, Biosense-Webster Inc., Diamond Bar, CA, USA or IBI CoolPath or Coolflex, Abbott, St Paul, MN, USA) was used for mapping and ablation, retro-aortic access to the LV was used as the primary method and transseptal technique as an alternative. An intracardiac echocardiography catheter (SOUNDSTAR, Biosense Webster, CA, USA) was delineated to create the ventricular 3-dimensional (3D) geometry and confirm catheter position and stability in VAs arising from the PM or MB area. A detailed activation map was obtained for each patient to define the earliest activation times during spontaneous or induced VAs, while pace mapping was also performed at the site of earliest activation. For VAs arising from the HPS, pace mapping may be ineffective for identifying the foci. Purkinje potentials preceding local ventricular activation were detected during both SR and VA in cases with an HPS origin.

The ideal target of RFCA was the earliest activation time and/or the best pace mapping (≥ 11/12 leads). Radiofrequency (RF) current was used as the energy source for ablation. The non-irrigated mode was used initially, and the irrigated mode would be choosed when need to achieve deeper lesions. Non-irrigated RF current was generally delivered in the temperature-controlled mode with a target temperature of 55 °C at a power of 30–50 W, while irrigated RF current was delivered in power-controlled mode at 30–45 W with an irrigation flow rate of 17–30 ml/min. If ventricular ectopy or VT was suppressed or terminated within the first 30 s of ablation, the additional current was delivered for 60–180 s. Otherwise, the RF delivery was terminated, and the ablation catheter was repositioned.

Acute success was defined as the absence of spontaneous or induced clinical VAs within 30 min (by intravenous administration of isoproterenol and programmed stimulation) after the last application of RF energy.

Procedure-related complications such as transient or persistent complete atrioventricular block, malignant arrhythmia, and cardiac tamponade, vascular access-related complication were recorded.

### Clinical follow-up

All patients were followed up via regular outpatient clinic visits and telephone calls after discharge. 12-Lead ECG and 24-h Holter monitoring were repeated at 1, 3, 6, and 12 months during the first year and every 6 months thereafter, or whenever the patient had symptoms suggesting VA recurrence.

Recurrence was defined as recurrences of sustained VT on 12-lead ECG, non-sustained VT and/or PVC burden > 5% on a 24-h Holter-ECG recording. The mid-term success of catheter ablation was defined as no recurrence of clinical VAs in the absence of anti-arrhythmic drug therapy until the end of follow-up.

### Statistical analysis

Statistical analyses were performed using the IBM SPSS Statistics for Windows, Version 24.0 (Armonk, NY: IBM Corp). Continuous variables are expressed as mean ± standard deviation. Categorical variables are expressed as frequency and percentage (proportion). The ANOVA was used to evaluate continuous data, and the chi-squared test or Fisher’s exact test was used to assess categorical data. Kaplan-Meier plots and log-rank tests were used for survival analysis. P < 0.05 was considered statistically significant.

## Results

### Patient characteristics

**Fig. 2:**
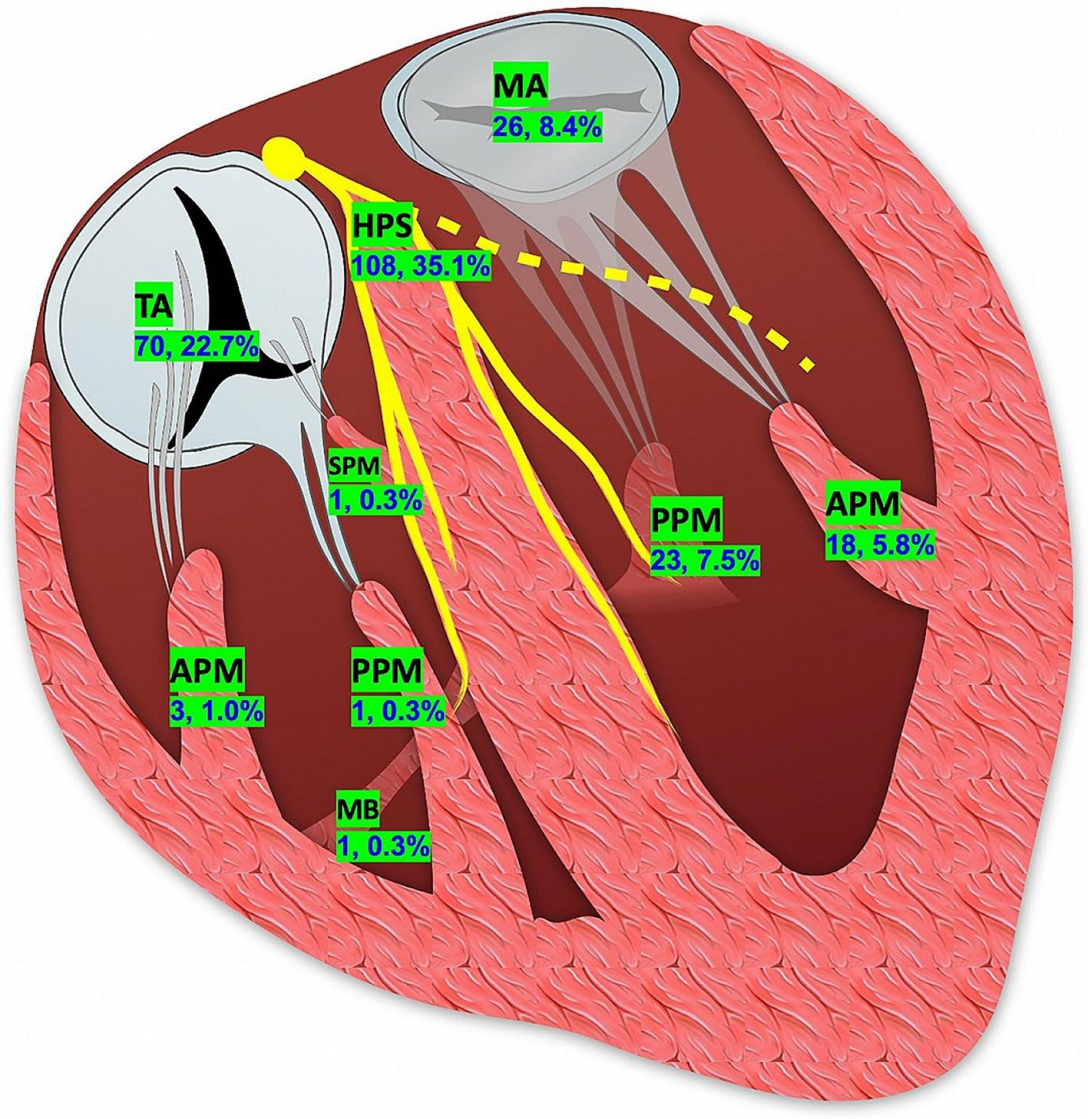
Schematic diagram of proportion and distribution of non-OTVAs. APM, anterior papillary muscle; HPS, His-Purkinje system; MB, moderator band; MA, mitral annulus; TA, tricuspid annulus; PPM, posterior papillary muscle; SPM, septal papillary muscle

The baseline characteristics of the study participants were summarized in Table 1. Sites of origin were defined as the HPS (n = 108), PM/MB (n = 47), TA (n = 70) and MA (n = 26) groups (Fig. 2). Patients in the HPS group were younger than the other groups, predominantly male (72.2%), and less often had comorbidities. Compared to the HPS group, patients in the PM/MB and MA groups had lower left ventricular ejection fraction (LVEF) (62.5 ± 5.1% and 60.8 ± 8.2%, vs. 64.7 ± 4.2%; *p* < 0.05). Patients in the HPS group had a higher prevalence of sustained VT, while PVC was the most common manifestation in the TA and MA groups.

**Table 1:** Baseline data

		HPS (*n* = 108)	TA (*n* = 70)	PM/MB (*n* = 47)	MA (*n* = 26)	*P*-value
**Age (year)**	33.8 ± 15.3		39.2 ± 19.6	43.5 ± 17.5	47.8 ± 18.9	<0.001
**Gender (male, %)**	78(72.2)		39(55.7)	29(61.7)	12(46.2)	0.033
**Hypertension, n (%)**	9(8.3)		17(24.3)	9(19.1)	5(19.2)	0.031
**DM, n (%)**	2(1.9)		3(4.3)	2(4.3)	4(15.4)	0.039
**Echocardiographic index**						
LVEF, %	64.7 ± 4.2		63.9 ± 3.1	62.5 ± 5.1	60.8 ± 8.2	0.017
LVDd, mm	46.9 ± 3.9		48.4 ± 4.8	48.3 ± 6.0	48.9 ± 4.9	0.072
LAD, mm	32.1 ± 4.5		33.5 ± 5.6	34.2 ± 4.8	34.4 ± 3.6	0.041
**AADs, n (%)**	76(70.4)		51(70.8)	35(74.5)	19(63.3)	0.745
**Clinical VA manifestation**						
Sustained VT, n (%)	74(68.5)		4(5.7)	8(17.0)	4(15.4)	<0.001
Non-sustained VT, n (%)	16(14.8)		6(8.6)	9(19.1)	3(11.5)	0.395
PVC, n (%)	23(21.3)		64(91.4)	36(76.6)	21(80.8)	<0.001
**PVC count, number/24 h**	29008.4 ± 41032.9		30694.7 ± 13979.2	38165.1 ± 29223.8	27736.3 ± 10661.1	0.027

HPS, his-purkinje system; TA, tricuspid annulus; PM, papillary muscles; MB, moderator band; MA, mitral annulus.DM, diabetes mellitus; LVEF, left ventricular ejection fraction; LVDd, left ventricular end-diastolic dimension; LAD, left atrial dimension; AADs, anti-arrhythmic drugs; VA, ventricular arrhythmia; VT, ventricular tachycardia; PVC, premature ventricular complex

### Procedural characteristics

At baseline, all patients had normal AH and HV intervals. Patients in the HPS group were significantly less likely to have spontaneous VAs compared to the other groups during the electrophysiological study. Catheter ablation of VAs arising from PM/MB was challenging, the total procedure time was significantly longer than other 3 groups. In the HPS group, one patient with a left posterior fascicular VT developed self-limiting ventricular fibrillation (VF) and vagal reflexes during the procedure, which was relieved by atropine injection. One patient in the TA group developed pericardial exudate during catheterization, which was immediately drained by pericardiocentesis, and no significant pericardial effusion was observed subsequently. Two patients developed transient third-degree AV block, and the PR intervals returned to normal during the procedure. No other procedure-related complications were observed. Acute success rates were 99.1%, 93.6%, 98.6%, and 92.3% in the HPS, PM/MB, TA, and MA groups, respectively, with no significant differences between them (Table 2).

**Table 2:** Procedural details

	HPS (*n* = 108)	TA (*n* = 70)	PM/MB (*n* = 47)	MA (*n* = 26)	*P*-value
**Spontaneous VAs (n, %)**	24 (22.6%)	51 (73.5%)	32 (68.8%)	16 (62.5%)	<0.001
**Activation time (msec)**	30.1 ± 18.4	29.2 ± 11.4	28.0 ± 9.4	26.3 ± 8.3	0.966
**Total RF delivered (s)**	232.9 ± 96.9	194.2 ± 99.1	297.8 ± 134.7	213.8 ± 118.8	<0.001
**Procedural complications (n)**	1	3	0	0	-
**Acute success rate (n, %)**	107 (99.1%)	69 (98.6%)	44 (93.6%)	24 (92.3%)	0.079

VAs, ventricular arrhythmias; HPS, his-purkinje system; TA, tricuspid annulus; PM, papillary muscles; MB, moderator band; MA, mitral annulus; RF, radio frequency

### Mid-term follow-up and clinical outcome

The follow-up lasted 4.38 ± 2.42 years, 15 (6.0%) cases including 10 in the HPS group (1 case died of advanced tumor), 1 in the PM group, and 2 each in the TA and MA groups were lost to follow-up.

**Fig. 3:**
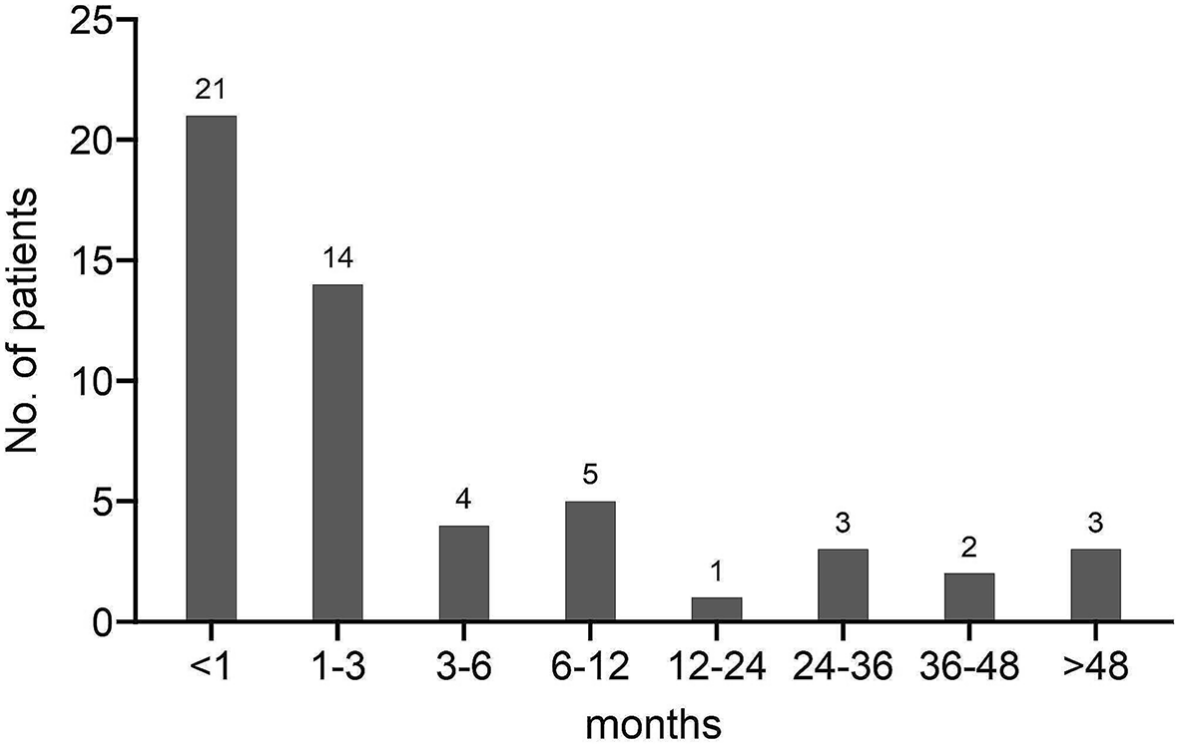
Time to first episode of VAs in all patients after the single catheter ablation

**Fig. 4:**
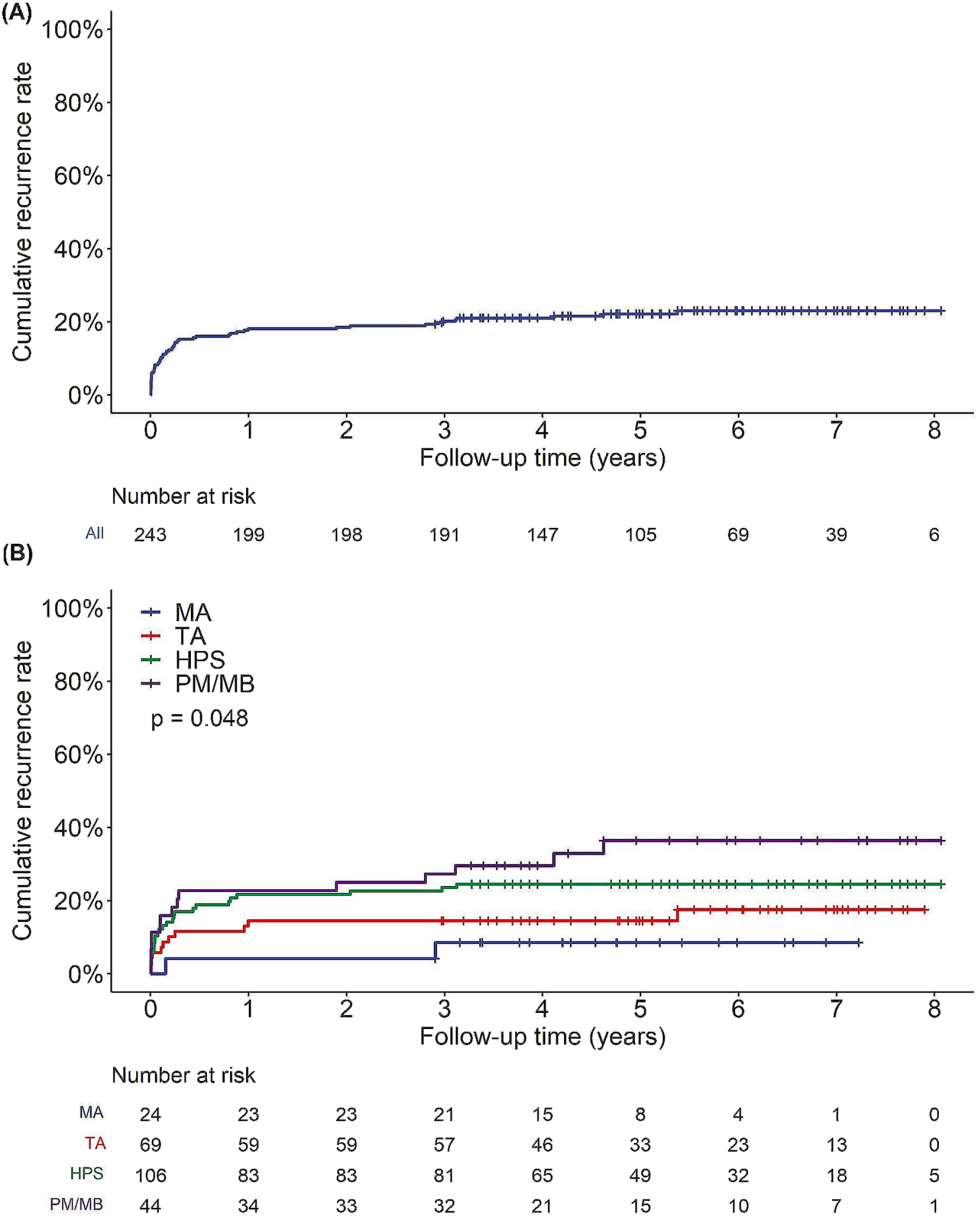
Kaplan-Meier analysis of recurrence of VAs after the single ablation in the overall patients (**A**) and among HPS, PM/MB, TA, MA groups (**B**)

284 RFCA procedures were performed on 251 patients, 244 (97.2%) patients achieved acute success. one each (0.9%, 1.4%%) in the HPS and TA groups, 3 (6.4%) in the PM/MB group, and 2 (7.7%) in the MA group failed during the single procedure. 53 (21.1%) patients including 25 (23.1%) in the HPS group, 15 (31.9%) in the PM/MB group, 11 (15.7%) in the TA group, and 2 (7.7%) in the MA group experienced VAs recurrence. Time of VAs recurrence of single procedure was within the first month in 21 (39.6%) patients, 1 to 3 months in 14 (26.4%) patients, 3 to 6 months in 4 (7.5%) patients, 6 to 12 months in 5 (9.4%) patients, later than 12 months in 9 (17%) patients. The distribution of recurrence is shown in Fig. 3. The single procedure success rate in all patients was 70.1%, in which 66.7%, 59.6%, 80%, and 76.9% were achieved in the HPS, PM/MB, TA, and MA groups, respectively (*p* = 0.284). With the Kaplan-Meier analysis, the recurrence rates of single procedure at 1 year and 3 years follow-up were 18% and 20%, respectively (Fig. 4A). There was a significant difference among the 4 groups in terms of the recurrence rate of single procedure (log-rank test, *p* = 0.048), The recurrence rate was significantly higher in the PM/MB group than in the MA (*p* = 0.023) and TA groups (*p* = 0.025) (Fig. 4B).

Among the recurrent cases, 21 (84%) patients in the HPS group underwent another 1 to 3 RFCA procedures, 2 (18%) patients in the TA group underwent another 1 to 2 RFCA procedures, and 5 (33.3%) patients in the PM/MB group underwent repeated RFCA procedure. 2 patients with recurrence in the MA group refused further ablation therapy. After multiple ablations, the overall success rate for non-OTVA patients was 76.5%. The mid-term follow-up success rates in the HPS, TA, PM/MB, and MA groups were 79.6%, 82.9%, 59.6%, and 76.9% respectively, and were significantly lower in the PM group than in the TA group (*p* = 0.035).

### Subgroup analysis of recurrence in the HPS and PM/MB groups

There were 74 patients (68.5%) with left posterior fascicular (LPF) VA and 19 (17.6%) with left anterior fascicular (LAF) VA in the HPS group. Acute success was achieved in 107 (99%) patients, representing a high success rate among these groups. One patient with left bundle branch VA failed in single procedure. During the follow-up period, there were 8 (42.1%) patients with LAF VA and 2 (50%) patients with inter-fascicular reentrant VT alone or in combination with bundle branch reentrant VT suffered recurrence (Table 3).

**Table 3:** Single RFCA procedure success and recurrence rates for HPS origin

Origin sites	All (n, %)	Immediate Success (n, %)	Recurrence (n, %)
LBB	8 (7.4%)	7 (87.5%)	1 (12.5%)
RBB	2 (1.9%)	2 (100%)	0 (0)
LAF	19 (17.6%)	19 (100%)	8 (42.1%)
LPF	74 (68.5%)	74 (100%)	14 (18.9%)
BBR-VT	1 (0.9%)	1 (100%)	0 (0)
IFR-VT	2 (1.9%)	2 (100%)	1 (50%)
BBR-VT + IFR-VT	2 (1.9%)	2 (100%)	1 (50%)

LBB, left bundle branch; RBB, right bundle branch; LAF, left anterior branch; LPF, left posterior branch; BBR-VT, bundle branch reentrant ventricular tachycardia; IFR-VT, intra-fascicular reentry ventricular tachycardia

In the PM/MB group, a total of 41 (87.2%) patients were found to be left ventricular PM origin; 18 of these cases were in the anterior left ventricular PM and 23 in the posterior left ventricular PM. Three of the 6 patients (12.8%) with right ventricular PM origin were located in the right ventricular anterior PM, the remaining 3 cases were in the right posterior PM, right septal PM, and moderator band (MB), respectively. Acute success was achieved in 44 (93.6%) patients. Three (6.4%) patients who failed the single procedure were all with the left ventricular PM origin. Fifteen (31.9%) patients suffered recurrence during the follow-up; of these cases, 13 (86.7%) with origin of the left ventricular PM (Table 4). No significant difference was observed in the recurrence rate of single procedure in patients with different VA origins within the PM/MB (log-rank test, *p* = 0.57) (Fig. S1).

**Table 4:** Single RFCA procedure success and recurrence rates for PM/MB origin

Origin sites	All (n, %)	Immediate Success (n, %)	Ineffective / Recurrence (n, %)
**Left ventricle, n (%)**	**41 (87.2%)**	**38 (92.7%)**	**3 (7.3%) / 13 (31.7%)**
LV—APM, n	18	16	2/6
LV—PPM, n	23	22	1/7
**Right ventricle, n (%)**	**6 (12.8%)**	**6 (100%)**	**0 (0) / 2 (33.3%)**
RV—APM, n	3	3	0/2
RV—PPM, n	1	1	0/0
RV—SPM, n	1	1	0/0
RV—MB, n	1	1	0/0

VA, ventricular arrhythmia; PM, papillary muscle; LV, left ventricle; RV, right ventricle; APM, anterior papillary muscle; PPM, posterior papillary muscle; SPM, septal papillary muscle; MB, moderator band

## Discussion

### Main findings

The goal of this study was to evaluate the outcome, safety, and efficacy of RFCA for idiopathic non-OTVAs. To the best of our knowledge, no previous study in the current literature has systematically investigated the effect of RFCA on non-OTVAs. The key findings are: (1) Idiopathic non-OTVAs accounted for 25.4% of all VAs. A total acute procedural success rate of the idiopathic non-OTVAs patients was 97.2% and the mid-term follow-up success rate was 76.5% after multiple procedures; (2) Time of non-OTVAs recurrence of the single ablation procedure was 1.69 (0.12, 9.72) months, 66% occurring within the first 3 months; (3) The single procedure success rates in the HPS, PM/MB, TA, and MA groups were 66.7%, 59.6%, 80%, and 76.9%, respectively, the recurrence rate was significantly higher in the PM/MB group than in the MA (*p* = 0.023) and TA (*p* = 0.025).

### Comparison with previous studies on non-OTVAs ablation

Previous studies mostly analyzed the ECG and electrophysiological characteristics of VAs at adjacent sites, and several studies compared their ablation success rates. Our study was the first to report the mid-term results of non-OTVAs ablation, including the sites of HPS, PM/MB, TA, and MA.

Sato et al. described the acute procedural success rates of TA and MA VAs were both 100% in all patients, and only one patient with VAs originating from TA experienced recurrence [5]. In the study by Li et al., after catheter ablation for 35 patients with VAs originating from TA, 3 recurrences occurred during a mean follow-up of 21 months, with a mid-term success rate of 91.43% [10]. In our study, as the cases with para-His origin were assigned to the TA and MA groups, the acute success rates of TA and MA VAs ablation were 98.6% and 92.3%, respectively, and the single procedure success rates were 80% and 76.9%, respectively, which were slightly lower to the results of previous studies. Good and colleagues found that, compared with the left branch system VAs, ablation of PM VAs required more energy and time, but had a lower success rate through 13 ± 11 months follow-up [7]. Huang et al. showed that the acute success rates of left PM and left posterior branch VAs were higher (100%) [8], but different degrees of recurrence were observed during follow-up. In the study by Li et al., the acute success rates after ablation of left inferior septal PM and left posterior branch VAs were 95.2% and 100%, respectively. Whereas, after 5–70 months follow-up, the recurrence rates were 40% and 4.7%, respectively [9]. In the present study, the acute success rate was 93.6% in the PM group, but the single procedural success rate and recurrence rate were 59.6% and 31.9%, respectively, as found in the mid-term follow-up, while the acute success rate and the recurrence rate were 99% and 23.1% in the HPS group, which were similar to the previous studies. The studies on fascicular VT (FVT), especially LPF origin, have shown that the long-term ablation success rate fluctuated from 70 to 90% [11, 12]. In our study, the single ablation procedure success rate of LPF origin was 81%, which was similar to previous studies. The incidence of BBR-VT accounted for 3.5-6% of all VT patients [13]. Our study included three cases of BBR-VT, but only one patient had a successful mid-term ablation, it can be presumed that BBR-VT is easy to relapse.

### Anatomical considerations, mapping and ablation approach

Mapping VAs originating from non-OT regions is challenging due to the complex anatomical structures. MA VAs are usually localized to the anterior, anterolateral and posterolateral aspects of the mitral valve, and TA VAs often arise from the septal portion (mostly from the His bundle region). In addition, in some cases, the origins of MA or TA VAs may possibly be located deep within MA or TA which were hard to ablate by conventional RFCA technology. If MA/TA VAs have failed ablation, positioning the ablation catheter at the ventricular edge of the MA/TA may result in successful arrhythmia elimination, as opposed to the above annulus approach [14, 15]. In our study, all of MA/TA VAs were firstly ablated by the below annulus approach. The PMs and MB are complex intracavitary structures, the special anatomic and variable sites of origin of VAs may increase the mapping and ablation challenge. In this study, 18 (38.3%) patients in the PM group experienced VAs relapse, and our findings agreed with previous literatures, patients with PM/MB VAs were still associated to higher clinical recurrence after multiple ablations. Recent researches reported that combine use of intra-cardiac echocardiography (ICE), multipolar catheters, cryo-catheter and CT image integration with 3D mapping system may be helpful for the ablation of PM-derived VAs [16–18]. Fascicular VT (FVT) is the most common form of idiopathic non-OTVAs. Left posterior FVT is more common than the anterior and septal FVT [19]. A number of studies have reported that the success rates of ablation of FVT are comparable to OTVAs [11, 12, 20]. So, RFCA is therefore recommended as the first-line therapy for fascicular PVCs/VT.

### Indication and recommendations for non-OTVAs ablation

In general, catheter ablation is preferred for patients with symptomatic idiopathic OTVAs and fascicular PVCs/VT due to their higher success rate and lower risk of complications. However, the detailed information for other forms of idiopathic non-OTVAs is limited and mostly restricted to the acute success rate of catheter ablation, for example, PM VAs, is lower and associated with more recurrences. In addition, access to and ablation at specific areas (e.g. epicardium, His bundle) may increase the risk of complications during the procedure. Up to now, the level of recommendation for ablation is low (*Class* IIa, Level C) from the latest VAs guideline [21]. But it’s worth noting that in patients with a cardiomyopathy suspected to be caused by frequent monomorphic PVCs, regardless of the origin, catheter ablation is highly recommended (*Class* I, Level C).

### Limitation

This study has several limitations. First, the study was a retrospective study involving a small number of patients, therefore, referral bias may exist. Second, in some cases, especially those in which the VA originated in the PM and MB, some useful mapping tools, such as ICE and steerable sheaths were also not routinely used, which may affect the success rate of the procedure. Furthermore, cases of non-OTVAs originating in the ventricular free wall, apex, and epicardium were not included. A larger sample size or multicenter study may be required to clarify the effect of catheter ablation on non-OTVAs.

## Conclusion

In our study, the idiopathic non-OTVAs originating from HPS, PM/MB, TA and MA accounted for 20% of all VAs. Acute ablation success can be achieved in majority of non-OTVAs, although a proportion of recurrences were observed in the mid-term follow-up, catheter ablation for idiopathic non-OTVAs was remain feasible and effective.

### Electronic supplementary material

Below is the link to the electronic supplementary material.

**Supplementary Material 1**: Figure S1. Kaplan-Meier analysis of recurrence of single procedure in patients with different VA origins within the PM/MB.

## Data Availability

The datasets used and analyzed during the present study are available from the corresponding author on reasonable request.

## Declarations

### Relationships with Industry and other entities

No disclosure was reported.
