# Supplementary material for: Mid-term outcome of catheter ablation of idiopathic non-outflow tract ventricular arrhythmias
Source: BMC Cardiovasc Disord. 2024 Jan 8;24:37. doi: 10.1186/s12872-023-03702-0 (PMC10775500; doi:10.1186/s12872-023-03702-0)
Supplement: Supplementary file 1 — Supplementary Material 1: Figure S1. Kaplan-Meier analysis of recurrence of single procedure in patients with different VA origins within the PM/MB. [file 12872_2023_3702_MOESM1_ESM.docx]

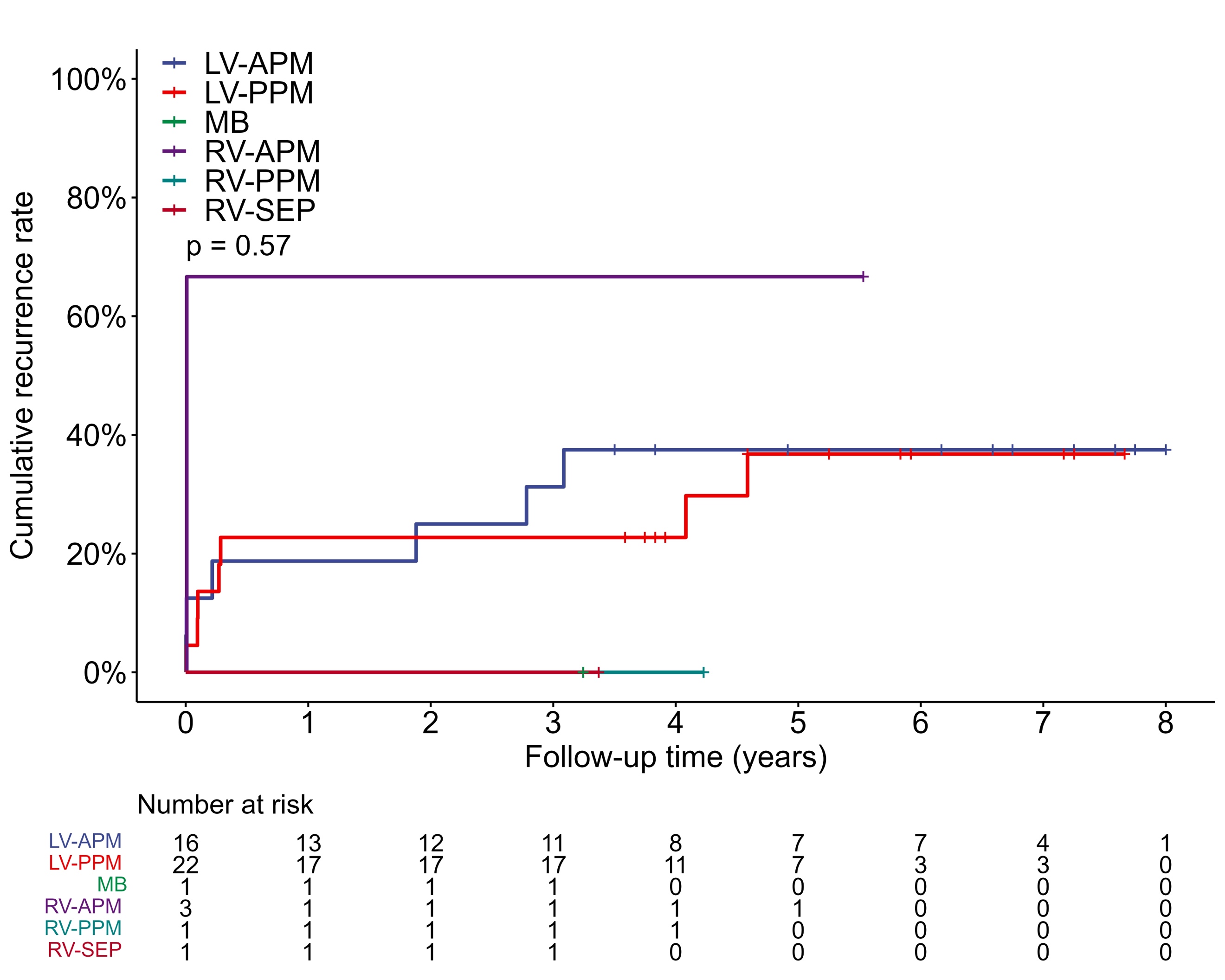


**Figure S1**. Kaplan-Meier analysis of recurrence of single procedure in patients with different VA origins within the PM/MB.
